# Unveiling Realities: Exploring and Uncovering Young Adults’ Views on Navigating Confidentiality and Disclosure in Healthcare Settings in Bangalore, Southern India

**DOI:** 10.7759/cureus.46158

**Published:** 2023-09-28

**Authors:** Andrea N Dias, Shirin Laturkar, Jeevitha K M, Anand D Meundi

**Affiliations:** 1 Surgery and Public Health, Shri Atal Bihari Vajpayee Medical College and Research Institute, Bangalore, IND; 2 Anesthesiology and Public Health, Shri Atal Bihari Vajpayee Medical College and Research Institute, Bangalore, IND; 3 Data Science, Prasanna School of Public Health, Manipal Academy of Higher Education, Udupi, IND; 4 Community Medicine, Dr. Chandramma Dayananda Sagar Institute of Medical Education and Research, Dayananda Sagar University, Bangalore, IND

**Keywords:** doctor-patient interaction, young adults, parent, guardian, confidentiality

## Abstract

Introduction

Research on the healthcare available to young adults in India is negligible. There is little to no data available to describe the Indian young adults' knowledge and attitude toward a doctor-patient interaction and their perceptions on what might be a barrier to confidentiality. Young adults in India, often face the unique dilemma of being old enough to make their own medical decisions and yet often finding themselves without the freedom or knowledge to do so. Understanding factors that young adults perceive to be affecting confidentiality and a barrier in their healthcare checkups can greatly improve the quality of healthcare provided to them.

Objective

The objective of the study was to assess knowledge and attitudes among young adults in Bangalore City regarding the maintenance of confidentiality by a doctor while seeking healthcare and to identify perceived factors the young adults believed to be affecting confidentiality and information disclosure when seeking healthcare.

Method

A cross-sectional, descriptive study was carried out using multi-stage random sampling. Four colleges were randomly selected from four geographic zones in Bangalore City (North, East, South, and West).

The investigators developed a 30-question questionnaire, comprising sections on patient details, perceptions regarding confidentiality, factors influencing history disclosure, etc., which was validated by a panel of four faculty members from one para-clinical and two clinical departments each, belonging to the investigators' medical college.

Subsequently, a pilot study with 33 participants was conducted and a sample size of 60 was arrived at assuming an 83.87% favorable knowledge regarding the right to refuse to disclose information to a doctor, with a 95% confidence interval and a 10% absolute allowable error. Further validation was done following the pilot study.

Student lists from the chosen colleges were obtained, and the required sample size was distributed based on probability proportional to size (PPS): 19, 19, 12, and 10 participants from the respective colleges. Random number tables were utilized to select the required number of participants from the student population. The participants of the pilot study were not included in the study. The questionnaire was administered digitally by the investigators, and in cases where a student declined to participate, an alternative participant was chosen using random number tables.

Results

Results demonstrate that 21.7% (13) of respondents were unaware that a doctor is legally bound to keep details of the visit confidential. A total of 93.3% (56) of the respondents report that a parent/guardian plays an active role in their doctor’s visit. Only 16.7% (10) of respondents strongly agreed that they felt comfortable enough to have an honest conversation with their doctor. Respondents report that they were most likely to withhold history regarding sexual practices (55%), alcohol use (35%), and smoking (31.7%).

Conclusion

Healthcare providers should take all possible measures to ensure confidential and quality care to the vulnerable young adult population. Breach of confidentiality, often in the form of a parent or guardian being present during the history-taking process. can be a barrier to building good rapport and negatively impact the doctor-patient relationship.

## Introduction

Young people are a diverse group with specific needs when it comes to their health and well-being [1]. Young adulthood ages approximately 18 to 26 is a critical time in life [2]. Although considered a relatively healthy demographic group, during this period, a high degree of exploratory behaviors are common as part of the process of establishing and developing self-identity. Meanwhile, the cognitive capacity for balanced reasoning is not yet fully developed. For these reasons, adolescence and young adulthood are characterized by increased likelihood of engaging in risky behaviors such as illicit drug use, binge drinking, and unprotected sex [3]. Efforts to provide preventive care to young adults are complicated by the fact that navigating the healthcare system during the transition from pediatric to adult providers is confusing and difficult [4].

Confidentiality protections are critical in the provision of comprehensive primary care for young adult patients [5]. Existing literature suggests that actual or perceived limits to confidentiality could influence the decisions of young adults about whether, and where, to seek care [2]. Indifferent treatment of patients and lack of patient privacy are common, yet are rarely acknowledged by traditional quality assessment methods [6]. When concerned about privacy, young adults are less likely to communicate openly with healthcare providers, particularly about issues related to substance use, mental health, and sexual behaviors, which influences information exchange about key health issues for this age group. Privacy concerns also influence these young adults' willingness to receive services such as pelvic examinations and testing for STIs or HIV, which should be a part of routine care for many youth [7]. Furthermore, qualitative analysis reveals that youth worry about privacy and may lie about their risk behaviors or not seek healthcare when concerned about confidentiality [8]. 

Young adults often forgo important sensitive services when they face a breach of confidentiality, which most likely occurs when their parents have access to the adult child's health information [9]. The presence of "informal" healthcare advocates during physician visits represents a unique privacy challenge [10].

Young adults should have access to developmentally appropriate high-quality confidential healthcare within professional, ethical, and legal guidelines. Clinicians and healthcare systems who provide this care have a higher likelihood of being able to effectively address the types of sensitive health issues linked to major causes of morbidity and mortality in this age group. These include health issues related to sexual behaviors, substance use, and mental health. Strategies to facilitate the ability to provide confidential healthcare include explaining its importance to parents and youth, routinely spending part of each visit alone with the patient, discussing confidentiality, and addressing issues that increase the risk of unintentional disclosure of protected information [11].

The primary objective of this study was to assess the knowledge and attitude of young adults in Bangalore city regarding doctor-patient confidentiality in seeking healthcare. Additionally, the study aimed to identify the perceived factors affecting confidentiality and information disclosure when seeking healthcare among these young adults.

## Materials and methods

A cross-sectional, descriptive study was carried out using multi-stage random sampling. Four colleges were randomly selected from four geographic zones in Bangalore city (North, East, South, and West). All medical colleges and other healthcare-associated colleges were excluded.

The investigators developed a 30-question questionnaire, comprising sections on patient details, perceptions regarding confidentiality, factors influencing history disclosure, etc. (multiple choice questions). A panel of four faculty members from one para-clinical and two clinical departments each, belonging to the investigators' medical college performed face validation and content validation.

Subsequently, a pilot study with 33 selected participants was conducted to calculate the required sample size. Assuming an 83.87% favorable knowledge regarding the right to refuse to disclose information to a doctor with a 95% confidence interval and a 10% absolute allowable error with a sample size of 60 participants was reached. Necessary changes were made to the questionnaire based on the feedback received by the participants of the pilot study. Another round of face and content validation was done following which the questionnaire was administered to the participants of the study.

Student lists from the chosen colleges were obtained, and the required sample size was distributed based on probability proportional to size (PPS). This resulted in the selection of 19, 19, 12, and 10 participants from the respective colleges. Random number tables were utilized to select the required number of participants from the student population. The participants of the pilot study were not included in the study. The questionnaire was administered digitally by the investigators, and in cases where a student declined to participate, an alternative participant was chosen using random number tables. 

Data obtained from the study were managed using MS Excel (Microsoft Corporation, Redmond, Washington), and data analysis was performed using R software. The study obtained approval from the Institutional Ethical Committee, i.e., the Ethics Committee of Bowring and Lady Curzon Medical College and Research Institute, approval no. BLCMCRI/IEC/RP/105/2021-22.

## Results

Sociodemographic characteristics

Among 60 respondents, 31 (51.7%) of respondents were male, and (29) 48.3% were female. The age group of respondents ranged between 18 and 25, with the majority of respondents aged 21 (45%), 20 (35%), and 22 (10%) years. The majority of respondents were heterosexual (86.7%) and unmarried (96.7%). They were largely Hindu (53.3%), Christian (25%), and Muslim (10%). Of the respondents, 36.7% were sexually active, 43.3% were not sexually active, and the remaining 18.3% preferred not to say (Table 1).

**Table 1:** Demographic details

Demographic characteristic	n (%)
Gender	
Male	31 (51.7)
Female	29 (48.3)
Age	
18	0 (0)
19	5 (8.3)
20	21 (35.0)
21	27 (45)
22	6 (10)
23	0 (0)
24	0 (0)
25	1 (1.7)
Sexual orientation	
Heterosexual	52 (86.7)
Homosexual	1 (1.7)
Bisexual	1 (1.7)
Pansexual	1 (1.7)
Asexual	3 (5.0)
Prefer not to say	2 (3.3)
Marital status	
Unmarried	58 (96.7)
Married	1 (1.7)
Prefer not to say	1 (1.7)
Level of education	
College - first year	1 (1.7)
College - second year	14 (23.3)
College - third year	38 (63.3)
College - fourth year	7 (11.7)
Level of education of father	
Graduate	28 (46.7)
Profession or honors	27 (45.0)
Intermediate or diploma	4 (6.7)
not formally educated	1 (1.7)
Level of education of mother	
Graduate	29 (48.3)
Profession or honors	23 (38.3)
Intermediate or diploma	5 (8.3)
Not formally educated	3 (5.0)
Religion	
Hindu	32 (53.3)
Christian	15 (25.0)
Muslim	6 (10.0)
Jain	1 (1.7)
Atheist	2 (3.3)
Agnostic	3 (5.0)
Prefer not to say	1 (1.7)
Personal income	
None	38 (63.3)
Less than Rs. 25,000/month	5 (8.3)
More than Rs. 25,000/month	9 (15.0)
Prefer not to say	8 (13.3)
Sexually active	
yes	22 (36.7)
No	26 (43.3)
Prefer not to say	11 (18

Knowledge of confidentiality during doctor visits

When knowledge of laws regarding doctor-patient confidentiality was assessed, 21.7%(13) of respondents did not know doctors are legally required to keep visit details confidential. 30%(18) were unaware they can refuse to disclose information (Table 2). Only 61.3%(19) of males knew about doctor-patient confidentiality, compared to 96.6% (28) of females (p=0.0012). Likewise, 64.5% (20) of males knew they could withhold information, while 75.9% (22) of females were aware (Table 3).

**Table 2:** Perceived knowledge of respondents regarding confidentiality

Question	Yes n (%)	No n (%)	Total n (%)
Are you aware that a doctor bound by law keeps details confidential?	47 (78.3)	13 ( 21.7)	60 (100)
Are you aware that you may refuse to reveal information to doctor?	42 (70)	18 (30)	60 (100)

**Table 3:** Comparison of perceived knowledge of confidentiality between males and females

	Male			Female		
Question	Aware n (%)	Not aware n (%)	Total n (%)	Aware n (%)	Not aware n (%)	Total n (%)
Are you aware that a doctor bound by law keeps details confidential?	19 (61.3)	12 (38.7)	31 (100)	28 (96.6)	1 (3.4)	29 (100)
Are you aware that you may refuse to reveal information to doctor?	20 (64.5)	11 (35.5)	31 (100)	22(75.9)	7 (24.1)	29 (100)

Involvement of parent/guardian in doctor’s visit

**Figure 1:**
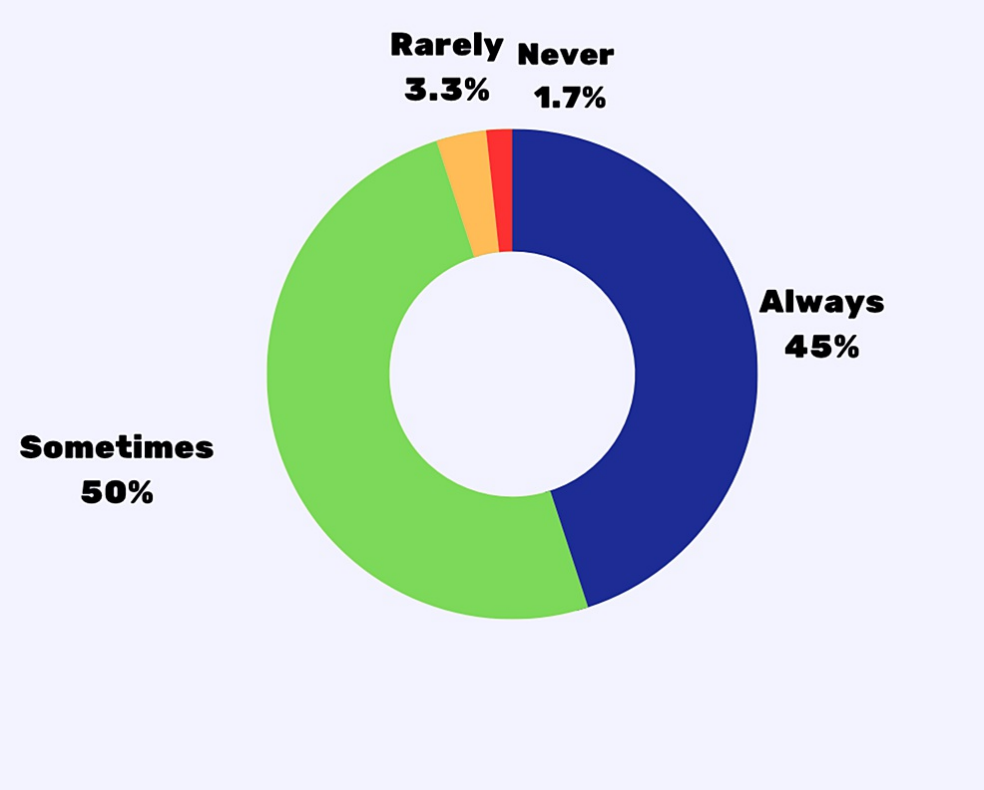
Parental/guardian accompaniment for medical appointments

**Figure 2:**
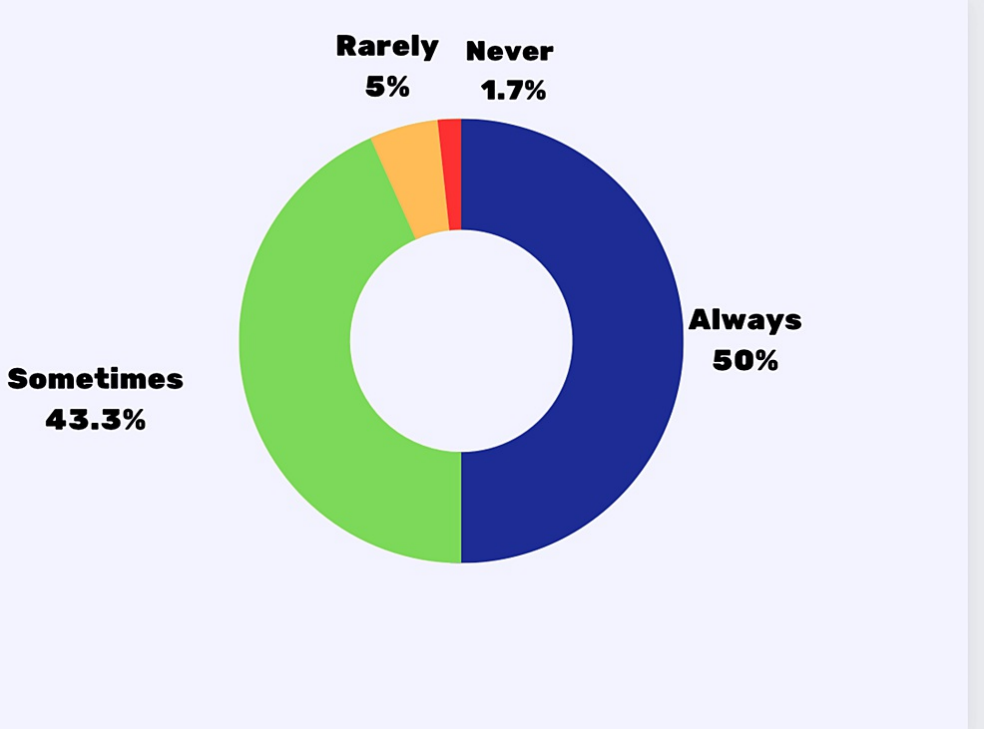
Parent/guardian's involvement during a medical appointment

In the study, only 5% (3) were rarely or never accompanied by parents for doctor's visits (Table 4, Figure 1). Most respondents' parents always (50%, 30) or sometimes (43.3%,26) played an active role (Table 4, Figure 2). 53.5% (32) restricted information due to a parent/guardian's presence (Table 4). 

**Table 4:** Parental/guardian involvement in medical appointments

	Always n (%)	Sometimes n (%)	Rarely n (%)	Never n (%)
Are you accompanied by a parent/guardian for a doctor's visit?	27 (45)	30 (50)	2 (3.3)	1 (1.7)
Is your parent/guardian active part of the visit?	30 (50)	26 (43.3)	3 (5)	1 (1.7)
Do you restrict the amount of information you give your doctor on account of your parents/guardian being present?	5 (8.5)	27 (45)	8 (13.3)	18 (30)

**Figure 3:**
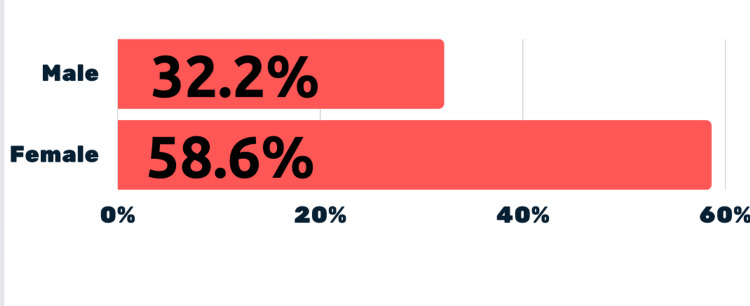
Comparison between males and females with regards to accompaniment of parent/guardian for a medical appointment

**Figure 4:**
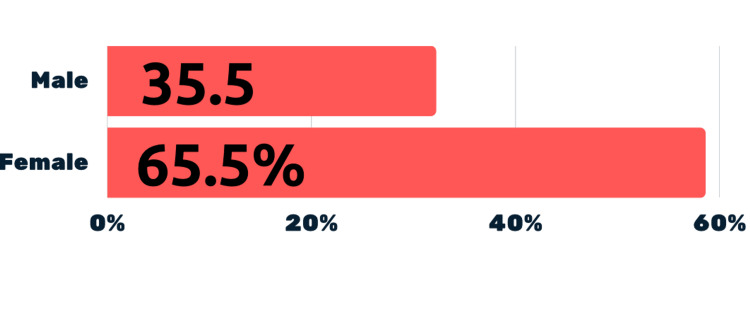
Comparison between males and females with regards to the involvement of parent/guardian in medical appointment

Comparative analysis showed that 32.2%(10) of males were always accompanied by a parent/guardian for medical appointments, while 58.6% (17) of females were (Figure 3). 35.5% (11)of males claimed parental involvement, compared to 65.5%(19) of females, nearly double the number (Figure 4).

Attitude of respondents regarding confidentiality during doctor visits

In assessing respondents' attitudes toward confidentiality during doctor visits, only 16.7% (10) strongly agreed to feel comfortable having open conversations with their doctor. 55%(33) agree they prefer going alone. Additionally, 61.7% (37) agree they would like consent before allowing parents/guardians to be part of the appointment (Table 5). 

**Table 5:** Respondents’ attitude regarding confidentiality during doctor visits

	Strongly agree n (%)	Agree n (%)	Neutral n (%)	Disagree n (%)	Strongly disagree n (%)	Total n (%)
I feel comfortable having an open and honest conversation with my doctor	10 (16.7)	20 (33.3)	26 (43.3)	3 (5)	1 (1.7)	60 (100)
I trust my doctor to keep details of my consultation confidential under all circumstances	11 (18.3)	28 (46.7)	19 (31.7)	2 (3.3)	0 (0)	60 (100)
I prefer going in for my appointment alone	14 (23.3)	19 (31.7)	19 (31.7)	7 (11.7)	1 (1.7)	60 (100)
I am comfortable with my parent/guardian being in the room during my doctor’s appointment	3 (5)	20 (33.3)	22 (36.7)	12 (20)	3 (5)	60 (100)
I feel confident/capable of going for my doctor’s appointment alone	16 (26.7)	24 (40)	15 (25)	4 (6.7)	1 (1.7)	60 (100)
I would prefer being asked for consent before allowing my parents/guardian to sit in for my appointment with me and be an active part of it	19 (31.7)	18 (30)	23 (38.3)	0 (0)	0 (0)	60 (100)
I would prefer a parent/guardian present for procedures that do not cause embarrassment	5 (8.3)	20 (33.3)	29 (48.3)	5 (8.3)	1 (1.7)	60 (100)
I possess the resources/knowledge to make an appointment without help from a parent/guardian	17 (28.3)	21(35)	17 (28.3)	5 (8.3)	0 (0)	60 (100)
I am satisfied with the medical care currently offered to me	9 (15)	31 (51.7)	18 (30)	1 (1.7)	1 (1.7)	60 (100)
I think better maintenance of patient confidentiality will help improve my consultation with the doctor	19 (31.7)	22 (36.7)	17 (28.3)	2 (3.3)	0 (0)	60 (100)

Practices regarding history disclosure

**Figure 5:**
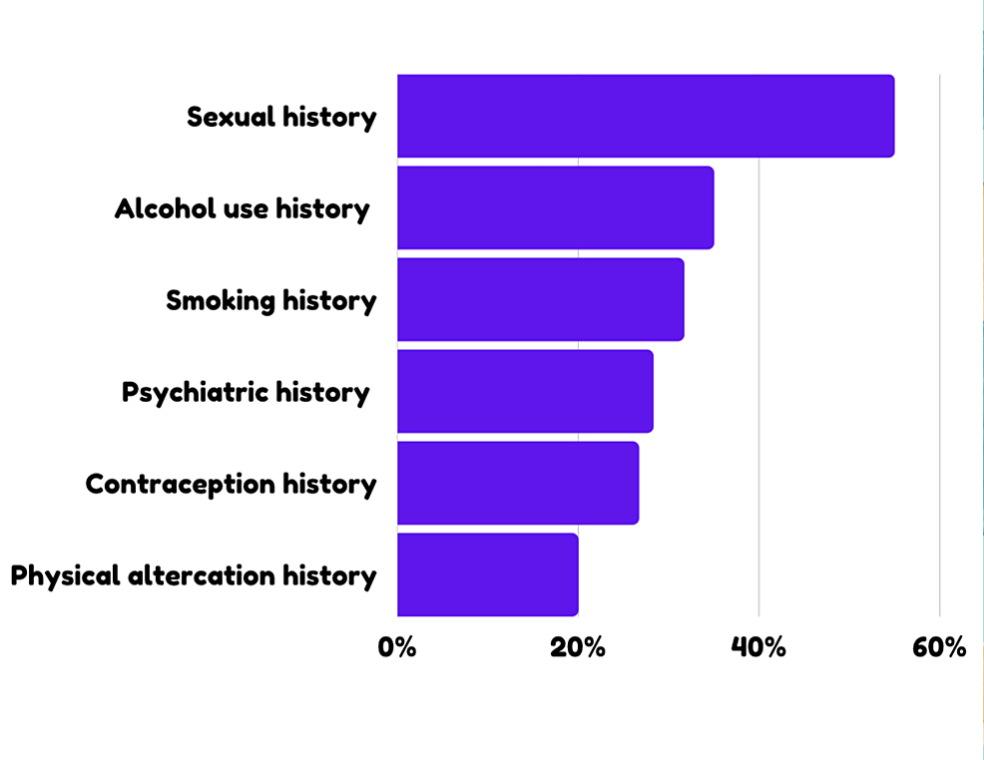
Medical histories most likely to be withheld from the physician by young adults

Results showed that respondents were most likely to withhold sexual history (55%), alcohol use (35%), and smoking (31.7%). Among males, sexual history (64.5%, 20), alcohol use (41.9%, 13), and smoking history (41.9%, 13) were commonly withheld. Among females, sexual history (44.8%, 13), psychiatric history (37.9%, 11), and alcohol use history (27.6%, 8) were commonly withheld (Figure 5).

## Discussion

In a study by Stephanie A. Grilo et al. (2016), examining factors associated with private time and confidentiality discussions among 1,918 US adolescents and young adults (13-26 years old), slightly over half of young women reported ever having private time with their provider (55%) or discussing confidentiality (55%). Young men had significantly lower rates, with 49% reporting private time (p=0.046) and 44% discussing confidentiality (p<0.001) [12].

The study revealed that 21.7% (13) of young adults surveyed were unaware of doctors' legal obligation to maintain visit confidentiality. This highlights situations where young adults lack awareness of their rights within doctor-patient interactions. Lack of awareness of rights creates potential for exploitation, as rights protect individuals' dignity. Additionally, 30% (18) were unaware they could refuse to disclose information to their doctor, impacting issues of consent. This highlights situations where consent and information may be given without a sense of choice.

These findings indicate a gap in confidentiality and privacy discussions between healthcare providers and young adults, with grave implications for patient privacy and the healthcare system. 

The study findings reveal that a mere 16.7% (10) of the respondents strongly agreed that they felt comfortable engaging in open and honest conversations with their doctors. This glaring communication gap poses a significant threat to the reliability of the healthcare system, especially in developing countries like India, where the population of 18- to 23-year-olds alone is 1.48 lakh [13]. Enhancing patients' experiences of care is a cornerstone of healthcare quality [14]. In fact, the 2012 National Institute for Health and Care Excellence Patient Experience Guideline has identified "comfort" as one of the seven outcomes of a good patient experience [15].

Several factors including lack of privacy, fear of judgment, feelings of insecurity, embarrassment, and the perception of harsh scrutiny significantly impact the comfort of young adults when interacting with healthcare providers. These elements may hinder young adults' willingness to disclose vital information to their doctors. Notably, a 1999 study conducted by JD Klein et al. examined factors associated with access to care among adolescents, including gender, insurance coverage, and having a regular source of healthcare. The study revealed that while girls and boys were equally likely to visit the same health professional as their parents, girls were slightly more inclined to prefer a different provider. Moreover, girls were more likely to report feeling too embarrassed to discuss a problem with a healthcare professional compared to boys [16].

A more recent study by Antionette Davey et al. (2013) explored the needs and experiences of young adults regarding healthcare services, aiming to identify reasons for lower satisfaction. The findings indicated that familiarity with the doctor played a significant role in the ease of communication and the young adults' confidence in providing relevant information. The study also revealed that young adults preferred having a single physician, and pre-existing personal relationships, such as blood relatives or family friends, often negatively impacted their comfort during visits to the physician [17]. To address this issue, healthcare providers should consciously reaffirm that the care provided will be completely confidential and professional. By creating an environment where young adult patients feel at ease and genuinely listened to, healthcare professionals can positively influence the doctor-patient interaction. This, in turn, encourages young adults to disclose their medical history with greater confidence, ultimately leading to improved diagnostic accuracy and healthcare outcomes [17].

In a study by Bradley E. Iott et al. (2019) on trust and privacy in doctor-patient interactions, a significant association was found between trust in confidentiality and patients withholding information from their doctors [18]. Surprisingly, the current study reveals that only 18.3% (11) of the respondents strongly agreed to trust their doctors to keep the details of their visit confidential. Bora Kim et al. (2017) propose that trust and emotional safety play a crucial role in communicating with young clients during healthcare visits, promoting open and engaging communication [3].

The 1999 study by JD Klein et al. further found that nearly one-third of the 6,748 adolescents surveyed had missed necessary care, with the most common reason being the reluctance to tell their parents (35%). Girls were more likely than boys to miss care (29% vs. 24%). Additionally, confidential visits varied by ethnicity, with Asians having the lowest rate of private time with physicians (49%) [16].

In this study, 55% (33) of the respondents either agreed or strongly agreed that they preferred to go for their doctor's appointment alone. Interestingly, 45% (27) stated that they are always accompanied by their parents or guardians, and 50% (30) said they are sometimes accompanied. Although parents or guardians may have good intentions, their presence during a doctor-patient interaction presents a confidentiality challenge. Older generations often believe it is their right and duty to be present and make medical decisions for their children, which can infringe on young adults' privacy. Going along with this dynamic may give the false impression of support but leaves young adults feeling neglected and apprehensive. Bora Kim et al. (2017) emphasize the importance of including young clients and promoting autonomy during health communication [3]. Healthcare providers must find a balance between the needs of young adults and the involvement of their parents or guardians.

Data also reveals a clear disparity between male and female respondents. Twice as many females are always accompanied by a parent or guardian compared to males (58.6% vs. 32.2%). Similarly, a higher percentage of females report their parents or guardians being actively involved in the doctor's visit (65.5% vs. 35.5%) compared to males. While it may be argued that the presence of an attendant is a legal and ethical requirement when examining female patients, it does not justify the presence of a guardian or parent during the history-taking process.

Data from the National Survey of Family Growth 2016 highlights that females aged 15-17 and 18-25 who had concerns about seeking sexual or reproductive healthcare due to potential parental discovery were less likely to receive such services compared to those without such concerns [19]. Alone time with the physician is crucial for young adult females, enabling them to discuss sensitive topics such as sex, contraception, pregnancy, abortions, and STIs. The presence of a parent or guardian, while well-intentioned, can hinder these discussions leading to unanswered healthcare concerns. This ultimately may cause patients to seek uncertain and potentially risky solutions from the internet or peers.

According to the Global Burden of Diseases, Injuries, and Risk Factors Study (GBD) 2019, alcohol-attributable burden ranks as the second-highest risk factor contributing to disability-adjusted life years among adolescents and young adults aged 10-24 years (GBD 2019 Risk Factors Collaborators, 2020). In a systematic review titled "Alcohol use in adolescents in India," Nadkarni et al. found that 55.3% of college-going students (aged 17-21 years) believed there was no risk of harmful effects from alcohol. A higher percentage of females compared to males held this belief (69.4% vs. 43.4%). The study also highlighted associations between alcohol consumption and tobacco use, illicit drug use, symptoms of attention deficit hyperactivity disorder (ADHD), suicidal thinking, planning, and attempts, as well as non-contact sexual abuse and perpetuation of violence [20].

In a 2002 study titled "Adolescent reports of Physician counseling for smoking," Catherine M Alfano et al. emphasized the need for more intensive interventions delivered by healthcare providers. They suggested that efforts should focus on helping providers accurately identify smoking habits and effectively communicate prevention or cessation messages. Special attention should be given to boys, experimental smokers, and youths with chronic health conditions, as their lower willingness to disclose smoking behaviors may hinder identification and intervention [21].

The study "Mental health help-seeking behaviors in young adults" compared access to mental healthcare across different age groups in Europe. It revealed that while three-quarters of psychiatric disorders in adults emerge before the age of 25, participants aged 18-24 were the least likely to seek care for mental health problems. The study also highlighted a UK survey where 35% of young adults experiencing current emotional or mental health difficulties did not seek any formal or informal help. Barriers to accessing care and support included perceived stigma, difficulty expressing concerns, accessing help, and a preference for self-reliance [22].

The respondents in this study reported being most likely to withhold information regarding sexual practices (55%), alcohol use (35%), and smoking (31.7%). A comparative analysis revealed that males were more likely to withhold sexual history (64.5%,20), alcohol use (41.9%,13), and smoking history (41.9%,13), while females were more likely to withhold sexual history (44.8%,13), psychiatric history (37.9%,11), and alcohol use history (27.6%,8). These findings highlight the high prevalence of alcohol use, smoking, high-risk sexual behaviors, and poor mental health among young adults. Paradoxically, these are the specific histories that young adults are most reluctant to share with their healthcare providers. This disparity raises important questions about the underlying reasons. Such information is crucial for physicians, not only as important contextual information but also as an opportunity to address risk behaviors, knowledge gaps, and discrepancies in health-related information, enabling the provision of holistic, preventive, and promotive healthcare.

The study conducted in 1997 by Ford CA et al. titled "Influence of Physician Confidentiality Assurances on Adolescents' Willingness to Disclose Information and Seek Future Health Care" demonstrated how assurances of confidentiality increased the number of adolescents willing to disclose sensitive information about sexuality, substance use, and mental health. The study showed an increase from 39% (68/175) to 46.5% (178/383) in disclosure rates (β=0.10, P=0.02) and an increase from 53% (93/175) to 67% (259/386) in the willingness to seek future healthcare (β=0.17, P<0.001) [23]. These findings underscore the importance of confidentiality assurances in promoting open communication and improving healthcare outcomes for young adults.

When adolescents seek healthcare, privacy concerns can significantly impact the quality of care they receive. Unfortunately, a substantial number of primary care physicians do not provide confidential health services to adolescents, discuss confidentiality with patients, or train their staff to provide accurate information about the confidential services available [7]. Encouraging health-related questions and answering them in a polite and neutral manner can help address these concerns.

In a 1997 study by Jonathan D. Klein et al., it was found that 30% of respondents preferred to have medical examinations without their parents present. Boys were less likely to prefer an examination without a parent present (19% vs. 41%), but they were more likely to have spoken with the doctor privately (62% vs. 53%). Older adolescents were also more likely to have had private conversations with their healthcare professionals (71% vs. 45%) [16].

Interestingly, in this study, it was found that 91.7% (55) of those who had a non-emergency personal procedure with a parent or guardian present did not feel significant embarrassment. The reasons for this difference are not fully explained, but it can be assumed that it is influenced by socio-cultural factors and differences in healthcare systems between the study populations. In countries like India, where confidentiality and privacy for young adults as patients are still developing concepts, access to confidential care is often restricted due to cultural and economic constraints. This practice is widely accepted as the norm in such contexts.

Research has shown that young adults are the least likely to respond to surveys about healthcare experiences and are generally less satisfied with the care they receive [18]. In this study, 61.7% (39) of respondents either strongly agreed or agreed that they were satisfied with the current medical care they received. However, 67.7% (41) also agreed or strongly agreed that better maintenance of patient confidentiality would help improve their consultations with doctors.

The study's limitations include a small sample size. The sample size was also restricted specifically to college students, i.e., an educated population leading to a possible respondent bias. A lack of previous studies conducted in India regarding the same led to the authors having to develop their own questionnaire and get a validation done for it. This validation, however, was done by faculty belonging to the investigators’ college thus leaving a scope for improvement. Subsequently, due lack of previous studies no comparisons of significance could be made.

## Conclusions

Educating young adults about their patient rights and effective utilization of healthcare services is essential to addressing their health needs. Establishing trust in physician confidentiality practices is crucial for building a good doctor-patient relationship, which ultimately leads to better treatment outcomes. While the current healthcare offered to young adults is not inherently dissatisfactory, there is room for improvement, especially regarding confidentiality in clinical settings. Strategies to enhance confidentiality include explaining its importance to parents and young adults, spending part of each visit alone with the patient, and addressing potential issues that may unintentionally disclose protected information. However, there is a lack of sufficient data on these specific subjects, particularly concerning young adults in India. Further research is needed to better understand the barriers young adults face during doctor's appointments and to identify potential solutions to improve the healthcare experience for this population.
